# Implementation of a Medication Reconciliation Process in an Internal Medicine Clinic at an Academic Medical Center

**DOI:** 10.3390/pharmacy6020026

**Published:** 2018-03-24

**Authors:** Kathryn M. Holt, Amy N. Thompson

**Affiliations:** 1 PGY2 Ambulatory Care Resident, Medical University of South Carolina, 280 Calhoun Street, QE 208, Charleston, SC 29425, USA; kathryn.holt@gmail.com; 2 Department of Clinical Pharmacy and Outcome Sciences, South Carolina College of Pharmacy-MUSC Campus, Charleston, SC 29425, USA

**Keywords:** interprofessional, medication reconciliation, discrepancies

## Abstract

Discrepancies in medication orders at transitions of care have been shown to affect patient outcomes in a negative way. The Joint Commission recognizes the importance of medication reconciliation through their National Patient Safety Goals, with an emphasis placed on maintaining accurate medication information for each patient. The primary objective of this study was to assess the effectiveness of implementing a medication reconciliation process in an internal medicine clinic at an academic medical center. A retrospective chart review of patients seen at an Internal Medicine Clinic within and Academic Medical Center, a continuity and teaching clinic for Internal Medicine residents and faculty practice clinic, was conducted. Nursing staff were educated by PharmDs to perform a standardized medication history during the triage process. Medication reconciliation data was analyzed for 3263 patients from 1 August 2014 to 27 February 2015. A total of 4479 discrepancies were found through this process, with the majority (71%) of discrepancies being medications on the list that patient was no longer taking. This project illustrated to our nursing and physician staff the need for regular thorough review of the patient medication list.

## 1. Background/Introduction

Discrepancies in medication orders at transitions of care have been shown to affect patient outcomes negatively, and have been shown to contribute to preventable medication errors and hospital readmissions [1,2]. The Joint Commission recognizes the importance of medication reconciliation through their Ambulatory Health Care National Patient Safety Goals, with an emphasis placed on maintaining accurate medication information for each patient. National Patient Safety Goal 03.06.01, regarding medication reconciliation when a patient is admitted to the hospital or seen in an outpatient setting, is “to maintain and communicate accurate patient medication information” [1].

Medication reconciliation, ‘the process of comparing medications a patient is taking with currently ordered medications; addressing duplications, omissions, and interactions; and identifying discrepancies,’ is an important part of each encounter a patient has with a healthcare provider. With the increasing prevalence of many ambulatory care chronic disease states and elaborate medication regimens has come the pressing need for improved healthcare collaboration with medication reconciliation to ensure patient safety and avoid negative patient-related events. A complete medication list can reduce drug-drug interactions, drug-disease interactions, other adverse drug reactions, and polypharmacy, as well as encourage interprofessional collaboration between healthcare professionals. Communication between healthcare providers and with patients ensures continuity of care [3].

A full medication reconciliation is considered a time-consuming and difficult process, which likely explains the lack of confidence and consistency performing these reconciliations [3,4]. Studies have shown that training members of the healthcare team to take a complete medication history improves accuracy and patient safety [5,6]. Additionally, by implementing a standardized medication reconciliation process and adequately training nurses and other staff, the medication reconciliation process has the potential to save time and improve physician, pharmacist, and nursing productivity [7].

The Joint Commission recognizes the difficulty in obtaining a complete and accurate medication list for patients, which is why a good faith effort at medication reconciliation qualifies as meeting the intent of the requirement listed in the Ambulatory Health Care National Patient Safety Goals [1]. Medication-related errors are one of the most preventable causes of patient harm, and medication reconciliation are vital to catch and resolve these problems in the healthcare setting [8].

## 2. Methods

A retrospective chart review of patients seen at an internal medicine (IM) clinic at a large academic medical center was conducted. This clinic is a continuity and teaching clinic for Internal Medicine residents and faculty practice clinic, serving an estimated 11,000 adult patients in an urban setting. There are approximately 17 attending physicians, 94 medical residents, 1 nurse practitioner, 1 physician assistant, 3 clinical pharmacy specialists, 8 licensed practical nurses (LPNs), 10 registered nurses (RNs), and 2 certified medical assistants (CMAs). The IM clinic sees an average of 225 patients on a given day, 80% of whom have at least one chronic disease process. Physicians in the IM clinic have historically maintained and updated medications within an electronic medical record using a variety of sources for medication information, including patient verbal history, pharmacy records, and vial verification. The IM nurses and pharmacists have not systematically been involved with the medication history or reconciliation process. 

Nursing staff were educated by PharmDs to perform a standardized medication history during the triage process. Educational sessions were held for nursing staff to improve both the medication reconciliation process as well as staff attitudes and expectations toward the project. Information provided in educational sessions included how to print a medication list, review the medication list, document medication discrepancies, and identify medications needing refills. Nurses were also given written instructions detailing this process. Each nurse then had a check-off session during which a PharmD observed and evaluated a direct patient interaction to ensure adequate skills to perform a medication history were obtained and consistency between nursing staff, this became a portion of the nursing staff annual competencies. The PharmD would provide specific feedback to the nurse after the medication history was complete, the PharmD would work with individual nursing members until both the pharmacist and the nurse were comfortable with the process. The review was performed annually. Information about the project and process for completing a medication reconciliation was provided to physicians during staff meetings weekly for residents and monthly for attendings.

During triage, nursing staff printed the patient medication list from the Epic Ambulatory^TM^ outpatient medical record and reviewed each medication with the patient. While this medical record allows for real-time updating to the record by the nursing staff, printing the medication list allowed for the PharmDs to easily assess for discrepancies found during the medication history process by the nursing team member. Based on patient verbal history or prescription vial verification when possible, the nursing staff documented “taking” or “not taking” for each medication both on the paper list and within the medical record. Discrepancies included completed therapies remaining on the medication list (including antibiotics), medications remaining on the list after therapy had been changed (i.e., a change in blood pressure medication due to side effect or ineffectiveness), dosing errors, duplications, or omissions. Discrepancies were noted on the medication list and given to the provider. Once the medication history was completed between the nurse and patient, the physician finalized the reconciliation process by addressing all discrepancies noted by the nursing staff and correcting the patient’s electronic medical record. After this process was complete, PharmDs retrospectively reviewed each medication list to note the errors and discrepancies found. The primary objective of this study was to assess the effectiveness of implementing a medication reconciliation process in the IM clinic. Secondary objectives included assessing interventions captured and degree of severity for errors caught, and to improve the medication reconciliation process itself, with the long-term goal of performing a thorough medication reconciliation on each patient seen in clinic.

## 3. Results

During the course of this project, each LPN, RN, and CMA was educated and had an observed patient medication history by the PharmD completed. This was done at the beginning of the project and completed annually as part of the annual competency assessment. Each new nursing team member went through education and check-off at the time of hire. Nursing in-services were provided four times over the course of the year to reinforce educational pieces. 

In the faculty practice clinic, data on 2700 patients revealed 3040 medications in the list that the patient was no longer taking, 462 duplications, 322 omissions, 251 dosing/frequency errors, and 200 finished courses of antibiotics (Table 1). In the resident continuity and teaching clinic, data on 563 patients revealed 306 medications patients were no longer taking, 29 duplications, 44 omissions, 25 dosing/frequency errors, and 13 finished courses of antibiotics (Table 2).

The most common type of error identified in patient charts at the IM clinic were medications a patient was no longer taking that remained on the medication list. Most frequently, these medications included expired narcotics, finished courses of antibiotics, or doses that had been changed but never deleted from the patient’s chart (i.e., an older dose of amlodipine remaining on the list after the dose had been increased). Significant discrepancies were identified in several instances. In one incident, the wrong medication was listed in a patient’s chart: the patient was taking insulin levemir, but insulin glargine was listed in the chart. In another instance, a complete medication reconciliation identified a patient who was not taking any of the 12 medications actively prescribed to them.

## 4. Discussion

While the benefit of pharmacist driven medication reconciliation process has been identified in several instances to be extremely effective, our clinic was in need of finding a method to identify and resolve discrepancies in the patient’s medication list [8,9]. Pharmacists within our clinic perform targeted medication reviews, particularly in the setting of hospital discharge but are not able to provide reviews for each patient seen. Working with clinic leadership, a pharmacist-led education process to allow for the triaging nursing team member to conduct a thorough medication review was implemented. The implementation of a nurse-driven medication history process has identified a large number of discrepancies in the patient charts within our clinic. The best practice or system in which to identify and resolve these discrepancies, however, is continuously evolving. Adequate and ongoing training is required to implement a meaningful medication reconciliation process, including assessment of physician compliance with completing the medication reconciliation process. The medication reconciliation process requires adherence to specific procedures by nursing staff, pharmacists, and physicians.

There was an improvement in the number of medication reconciliation performed by nursing staff, noted especially during the prime training months when the project was unveiled (September—November). The number of medication reconciliation performed compared with the number of patients seen in clinic per month, however, reflected an overall lack of compliance with the process. This lack of compliance could be multi-factorial, including the history not being completed by nursing staff and physicians not turning in medication lists for review. Additionally, a thorough medication reconciliation requires patient participation, which often presents challenges. The process requires patients to bring their medications to clinic, fill prescriptions regularly at one pharmacy, or know their medication list well. Given the complexity of some medication regimens and the number of prescriptions an average patient has on their profile; this process can be difficult. 

There were numerous limitations noted during implementation of this project. Due to the time-consuming nature of the medication reconciliation process implemented in the IM clinic, we encountered some pushback from both our nursing and physician colleagues. Having the nursing staff identify medications requiring a refill prior to the physician coming into the room, however, was found to be helpful with timeliness of the clinic, as it had the potential to reduce the number of phone calls after a visit requesting refills. Results were presented to nursing staff and physicians to try to increase staff awareness and enthusiasm for the project. In a busy clinic with a large number of patients seen daily, the medication reconciliation procedures we tried to implement are likely not sustainable. In the continuity and teaching clinic, residents rotate every month; so new residents were constantly entering the clinic. The frequent staff changes contributed to the difficulties implementing a sound system for medication reconciliation. In the Epic Ambulatory^TM^ outpatient medical record, clinicians reviewing the medication list have the option of clicking “mark as reviewed”, which will apply to all medications on a patient’s list, without the necessity of actually evaluating each medication individually. This technology made it difficult to determine whether a full medication reconciliation had been completed or simply marked as completed. Lastly, we were unable to capture data on all patients seen in the clinic. Our clinic sees an average of 225 patients a day, and we were able to capture roughly 12% of patients a month. While a medication review was performed by the nursing staff at each visit, often the paper documentation was not turned in at the end of the visit.

Through this project we identified a large number of medication discrepancies in the patients in our clinic. While there are standardized tools for performing a proper medication history on the inpatient side, such as the MARQUIS project [10], our team aimed to create a sustainable model for our busy general medicine clinic.

## Figures and Tables

**Table 1:** Faculty practice clinic.

Month	Number of Patients with Captured Data	Medications Patients Were No Longer Taking (%)	Duplications (%)	Omissions (%)	Dosing/Frequency Errors (%)	Finished Courses of Antibiotics (%)
August	284	253 (89%)	54 (19%)	26 (9%)	23 (8%)	12 (4%)
September	512	586 (114%)	74 (14%)	38 (7%)	42 (8%)	46 (9%)
October	551	642 (117%)	106 (19%)	68 (12%)	74 (13%)	41 (7%)
November	402	461 (115%)	94 (23%)	61 (15%)	28 (7%)	26 (6%)
December	470	438 (93%)	43 (9%)	32 (7%)	24 (5%)	26 (6%)
January	224	315 (141%)	47 (21%)	36 (16%)	30 (13%)	28 (13%)
February	257	345 (134%)	44 (17%)	61 (24%)	30 (12%)	21 (8%)

**Table 2:** Resident continuity and teaching clinic.

Month	Number of Patients with Captured Data	Medications Patients Were No Longer Taking	Duplications	Omissions	Dosing/Frequency Errors	Finished Courses of Antibiotics
August	36	9 (25%)	3 (8%)	1 (3%)	5 (14%)	2 (6%)
September	50	57 (114%)	5 (10%)	5 (10%)	4 (8%)	3 (6%)
October	330	206 (62%)	17 (5%)	22 (7%)	11 (3%)	6 (2%)
November	147	34 (23%)	4 (3%)	16 (11%)	5 (3%)	2 (1%)
